# Protein Phosphatase PP2C Identification in *Entamoeba* spp

**DOI:** 10.1155/2021/5746629

**Published:** 2021-10-16

**Authors:** Abril Navarrete-Mena, Judith Pacheco-Yépez, Verónica Ivonne Hernández-Ramírez, Alma Reyna Escalona-Montaño, Jenny Nancy Gómez-Sandoval, Mario Néquiz-Avendaño, Bibiana Chávez-Munguía, Emiliano Tesoro-Cruz, Patricia Talamás-Rohana, María Magdalena Aguirre-García

**Affiliations:** ^1^Sección de Estudios de Posgrado e Investigación, Escuela Superior de Medicina, Instituto Politécnico Nacional, Ciudad de Mexico 11340, Mexico; ^2^Unidad de Investigación UNAM-INC, División de Investigación, Facultad de Medicina, Instituto Nacional de Cardiología Ignacio Chávez, Ciudad de Mexico 14080, Mexico; ^3^Departamento de Infectómica y Patogénesis Molecular, Centro de Investigación y Estudios Avazados, CINVESTAV-IPN, Ciudad de México, CP 07360, Mexico; ^4^Universidad Politécnica del Valle de Toluca, Estado de Mexico 50904, Mexico; ^5^Unidad de Medicina Experimental, Facultad de Medicina, UNAM, Ciudad de Mexico 06726, Mexico; ^6^Unidad de Investigación Biomédica en Inmunología e Infectología, Hospital de Infectología, Centro Médico Nacional “La Raza”, IMSS, Ciudad de Mexico 02990, Mexico

## Abstract

*Entamoeba histolytica* is the causative agent of amoebiasis, and *Entamoeba dispar* is its noninvasive morphological twin. *Entamoeba invadens* is a reptilian parasite. In the present study, Western blot, phosphatase activity, immunofluorescence, and bioinformatic analyses were used to identify PP2C phosphatases of *E. histolytica*, *E. dispar*, and *E. invadens.* PP2C was identified in trophozoites of all *Entamoeba* species and cysts of *E. invadens*. Immunoblotting using a *Leishmania mexicana* anti-PP2C antibody recognized a 45.2 kDa PP2C in all species. In *E. histolytica* and *E. invadens*, a high molecular weight element PP2C at 75 kDa was recognized, mainly in cysts of *E. invadens*. Immunofluorescence demonstrated the presence of PP2C in membrane and vesicular structures in the cytosol of all species analyzed. The ~75 kDa PP2C of *Entamoeba* spp. shows the conserved domain characteristic of phosphatase enzymes (according to in silico analysis). Possible PP2C participation in the encystation process was discussed.

## 1. Introduction

*Entamoeba histolytica* is an anaerobic parasitic amoebozoan and the causal agent of amoebiasis, which manifests as gastrointestinal disorders (e.g., amoebic colitis and dysentery) and less commonly as extraintestinal ulcers (e.g., amoebic liver abscesses, purulent pericarditis, and cerebral amoebiasis) [1–3]. According to the World Health Organization, there are up to 100,000 deaths per year due to amoebiasis, representing a high mortality rate [4].

*E. histolytica* has a faecal-oral life cycle involving a trophozoite stage and a cyst stage, the first of which is motile and proliferative, while the second is infective. The cysts enter humans by means of infected food or water and pass through the digestive tract until reaching the ileum. There, cysts undergo the process of excystation, consisting of the emergence of immature trophozoites from the cysts and their migration towards the colon, where they mature and proliferate [5, 6].

Unfortunately, *E. histolytica* does not form cysts in vitro. In contrast, *E. invadens* has this capability which allows researchers to faithfully reproduce the events of the amoebic life cycle. *E. invadens* strain IP-1 is a reptilian parasite obtained from the tortoise *Chrysemys picta* and is pathogenic to snakes [7]. Since its life cycle is like that of *E. histolytica*, it is used as a model for studying the process of encystation. The genomes of *E. invadens* and *E. histolytica* are also remarkably similar. However, the genome of *E. invadens* is highly repetitive and is 38% longer than that of *E. histolytica* [8].

Two morphologically identical species were previously classified as *E. histolytica*. Biochemical, immunological, and genetic data have made it possible to distinguish these two species, *E. dispar* and *E. histolytica*, based on genetic, structural, and functional differences [4, 9]. *E. dispar* is a nonpathogenic and more prevalent species, while *E*. *histolytica* causes intestinal amoebiasis and liver abscesses [10]. The life cycle of *E. dispar* is like that described for *E. histolytica*, except that after excystation, the former is not able to break the intestinal mucosal barrier and consequently is not invasive. It remains in the colon and is considered commensal. Although *E. histolytica* has been found to penetrate the mucosal barrier, the stimuli responsible for this movement are not known. Likewise, the conditions engendering the encystation of *E. histolytica* have yet to be defined. The subpopulation of nonphagocytic trophozoites is approximately 40% for *E. dispar* and 5% for *E. histolytica* [10, 11].

The no pathogenicity of *E. dispar* can be explained by multiple characteristics: poor adhesion to red blood cells leading to reduced haemolytic activity, low expression of phosphatidylserine (involved in the process of phagocytosis) [11], and the absence of a CP5-type protease [12, 13]. The latter enzyme participates in the *E. histolytica* induced formation of amoebic liver abscesses [14]. Experimental infections in murine models have demonstrated the differential expression by *E. dispar* and *E. histolytica* of various factors modulating the development of the disease, including Gal/Gal Nac lectins, cysteine proteases, and amoebapores [15–17].

These two species also differ in the production and secretion of protein phosphatases, which are known to regulate the dephosphorylation (removal of a phosphate group) of some host cell amino acid residues, such as threonine, serine, and tyrosine [18]. Phosphorylated proteins play a crucial role in cell homeostasis and are regulated by protein kinases, enzymes responsible for adding phosphate groups to specific amino acids. *Entamoeba* secretes protein phosphatases into the culture medium [19] and has a membrane-associated protein phosphatase with protein tyrosine phosphatase (PTP) activity capable of breaking the HeLa cell actin cytoskeleton [20, 21]. Among the other PTPs in *E. histolytica* are *Eh*PTPA and *Eh*PTPB. Both these genes are differentially expressed in trophozoites from amoebic liver abscess [22]. *E. histolytica* also has a PRL phosphatase (a type of PTP) located in small patches on the outer membrane and in internal vesicles and is known to dephosphorylate tyrosine residues [23].

*E. histolytica* was recently reported to have many phosphatases (250), resulting in a phosphatome 1.3 times greater than that of human beings. This species contains 145 protein serine/threonine phosphatases: 79 PTPs, 18 phosphatases with endonuclease-exonuclease activity, and 8 pyrophosphatases. Only 18 are dependent on metals such as magnesium and manganese [24]. Among the latter group is protein phosphatase 2C (PP2C). There are no reports, to our knowledge, of studies on PP2C phosphatases in *Entamoeba* spp.

The aim of the present study was to identify possible PP2C serine threonine protein phosphatases in *E. histolytica*, *E. invadens*, and *E. dispar*.

## 2. Materials and Methods

### 2.1. Culturing the Parasites

*E. histolytica* trophozoites (HM1: IMSS) were cultured according to the Diamond Method [25], while *E*. *dispar* (SAW 760 strain) and *E. invadens* (IP-1; ATCC 30994) trophozoites were maintained in LYI-S-2 medium TYI-S-33 and incubated at 37°C and 28°C, respectively [26].

### 2.2. *E. invadens* Encystation

*E. invadens* trophozoites (IP-1 strain) were axenically grown at 28°C in complete LYI-S-2 medium to induce encystment, and trophozoites were harvested in the logarithmic phase of growth as previously described [27]. The amoebae were incubated at 28°C for 12, 24, and 48 h. Cysts were harvested from the encystation medium by sedimentation.

### 2.3. Obtaining Membrane and Cytosolic Fractions from Extracts of *Entamoeba* spp.

The trophozoites of *E. histolytica*, *E. invadens*, and *E. dispar* were grown until they became fully confluent and then harvested after the culture bottle was placed on ice for 10 min. The parasites were centrifuged at 486 x g at 4°C for 5 min, the growth medium was discarded, and the pellet was suspended and washed three times. Two washes were performed with phosphate-buffered saline (PBS), and the third wash was performed with 20 mM Tris (pH 7.8) that contained protease inhibitors (50 mM E-64, 10 mg/mL aprotinin, and 1 mM benzamidine), and this was followed by centrifugation as mentioned above. The parasites obtained were subjected to three freeze/thaw cycles from -70°C to 37°C. Subsequently, an aliquot of the extract, considered the total extract, was transferred to a new tube. The suspension was centrifuged at 20,500 x g at 4°C for 5 min. The resulting pellet, considered the membrane fraction, was suspended in lysis buffer. The supernatant contained the cytosolic fraction, modified from [16]. All fractions were frozen at -70°C to await further use.

### 2.4. Phosphatase Activity Assays

Acid phosphatase activity was determined as previously described [28]. Briefly, 1.0 *μ*g of the total extract, membrane fraction, or cytosolic fraction was placed in buffer (200 mM sodium acetate, pH 5, with 10 mM *p*-nitrophenyl phosphate (*p*-NPP) in a final volume of 100 *μ*L) and incubated at 37°C for 60 min. The reaction was stopped with 20 *μ*L of 2 N NaOH, and the absorbance was measured at 405 nm using a microtiter plate reader.

### 2.5. Effect of Phosphatase Inhibitors on the Membrane Fraction of *Entamoeba* spp.

The phosphatase activity in the membrane fraction was examined in the presence of specific PTP inhibitors (200 *μ*M sodium orthovanadate, 200 *μ*M ammonium molybdate, and 200 *μ*M sodium tungstate) and a specific PP2C inhibitor (200 *μ*M sanguinarine) [29, 30]. For the inhibition assays, 100 *μ*L of the reaction mixture was preincubated at room temperature for 15 min. After adding the p-NPP substrate, the solution was incubated at 37°C for 60 min, and the reaction was stopped with 20 *μ*L of 2 N NaOH. Absorbance was measured at 405 nm using a microtiter plate reader. All reagents were acquired from Sigma-Aldrich.

### 2.6. Production of Polyclonal Antiserum

Anti-*Lmx*PP2C antibodies were generated in rabbits following the procedure established [31]. Briefly, rabbits were injected intramuscularly with 135 *μ*g of recombinant *Lmx*PP2C emulsified in complete Freund's adjuvant, and the same procedure was repeated 2 weeks later without adjuvant. The immunization was based on two weekly intramuscular injections, after which the animals were bled, and antiserum was separated by centrifugation and stored at -20°C. Rabbits were housed at the animal facility of the Research Unit of Experimental Medicine of the Medicine Faculty, UNAM, and handled in accordance with the National Ethical Guidelines for Animal Health NOM-062-ZOO-1999 and the guidelines recommended for animal care by the institutional Ethics in Research Committee [30].

### 2.7. Western Blot Analysis

The fractions were resolved with 10% SDS-PAGE using 25 *μ*g each of the total extract, membrane fraction, and cytosolic fraction of *E. histolytica*, *E. invadens*, and *E. dispar*. The positive control consisted of 0.7 *μ*g *Lmx*PP2C recombinant protein. Gels were electrotransferred, and membranes of low fluorescence were blocked with Li-Cor blocking buffer at room temperature for 30 min. The gels were incubated with two specific anti-*Lmx*PP2C and antitubulin antibodies diluted 1 : 1000 in 0.1% Tween LiCor blocking buffer overnight at 4°C. The membranes were washed with PBS Tween and incubated with a secondary antibody, IRDye 680LT goat anti-rabbit IgG, and goat anti-mouse IRDYE 800 CW at a dilution of 1/10,000 for 1 h under gentle shaking. The membranes were washed three times with 1X PBS, and the proteins were examined on an Odyssey Infrared Imaging System according to manufacturer's instructions.

Likewise, Western blot assays were performed with the cytosolic fractions of *E. invadens* trophozoites and cysts, subjecting them to lysis with 20 mM Tris lysis buffer (containing 2.5% Triton X-100), as previously mentioned. Subsequently, they were lysed by freeze/thaw cycles with sonication before utilizing 20 *μ*g of protein in a 10% SDS-PAGE procedure, modified from [32].

### 2.8. Immunofluorescence Assays

#### 2.8.1. Trophozoites

Trophozoites of *E. histolytica*, *E. invadens*, and *E. dispar* were washed with PBS, adhered to coated slides for 15 min, and fixed with 4% paraformaldehyde in PBS at 37°C for 1 h. Slides were then washed three times with PBS. We used two different conditions, nonpermeabilized trophozoites and permeabilized trophozoites, and in the latter case, 0.01% Triton X-100 was used to permeabilize, followed by blocking with 10% foetal bovine serum (FBS) for 1 h. Parasites were then incubated with antibodies against the PP2C recombinant protein of *L. mexicana* at a dilution of 1 : 50 overnight at 4°C. After the appropriate washings, trophozoites were stained with the secondary antibody anti-rabbit IgG conjugated to TRITC at a dilution of 1 : 100. Microscope slides were mounted by using Vectashield with DAPI mounting medium (4′,6′-2 diamidi-no-2-phenylindole) and observed under a confocal microscope, modified from [33].

#### 2.8.2. Cysts

Upon reaching 12 or 24 h of encystation, cysts of *E. invadens* were washed with 1X PBS, fixed with 4% paraformaldehyde in PBS at 37°C for 1 h, and then, washed three times with PBS. We used two different conditions, nonpermeabilized and permeabilized cysts; in the latter case, 2.5% Triton X-100 was used to permeabilize, and then, cysts were blocked with 10% FBS for 1 h. The two groups of cysts (having undergone 12 or 24 h of encystation) were incubated with antibodies against the PP2C recombinant protein of *L. mexicana* (anti-*Lmx*PP2C) at a dilution of 1 : 50 overnight at 4°C, followed by washing and staining with the secondary anti-rabbit IgG antibody conjugated to TRITC (tetramethyl rhodamine) or FITC (fluorescein isothiocyanate) as necessary, at a dilution of 1 : 100. Slides were mounted using Vectashield with DAPI mounting medium (4′,6′-2 diamidi-no-2-phenylindole) and observed under a confocal microscope, modified from [34]. Secondary antibody was used as a negative control.

### 2.9. *In Silico* Analysis

Based on the Western blot results of the crossreaction of polyclonal serum (*αLmx*PP2C) during the encystation of *E. invadens*, a search was carried out in the AmoebaDB database. The criteria utilized were the amoebic species, molecular weight, and homology with the protein sequence of *L. mexicana* (Lmx_25.0750). Some physicochemical parameters were taken from the most likely sequence returned, as well as orthology and possible cellular function [35]. The cell location was determined with the WoLF PSORT tool [36]. Afterwards, orthologues of this sequence of *Entamoeba* were aligned with the sequence of PP2C of *L. mexicana* in the COBALT program [37]. To generate the cladogram, the sequence of *E. invadens* was aligned with all sequences of the members of the orthologue group. Subsequently, the best model for establishing the cladogram was obtained using the MEGA 7 program [38] and then edited on the iTOL platform [39]. The 3D model was predicted on the I-TASSER server [40], and the degree of evolutionary conservation of amino acids was evaluated on the ConSurf server. The model was visualized with the UCSF Chimaera program [41, 42].

### 2.10. Statistical Analysis

In all cases, differences were examined with Tukey's tests, Mann-Whitney *U* tests, and Student's *t*-tests.

## 3. Results

### 3.1. Phosphatase Activity in Trophozoites of *E. histolytica*, *E. invadens*, and *E. dispar* and Phosphatase Inhibitors

**Figure 1:**
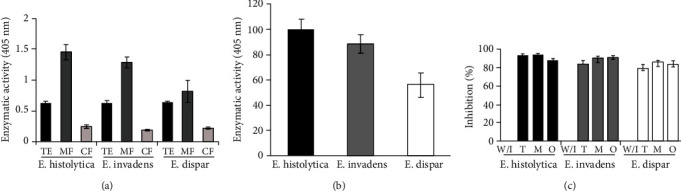
Phosphatase activity in *Entamoeba* spp. (a) Enzymatic activity of the total extract (TE, black bar), membrane fraction (MF, dark grey bar), and cytosolic fraction (CF, light grey bar) of *E. histolytica*, *E. invadens*, and *E. dispar*. (b) Relative value (considering *E. histolytica* as 100% percent) of phosphatase activity in the MF of *E. invadens* (dark grey bar) and *E. dispar* (white bar). (c) Phosphatase activity of the MF of trophozoites from *E. histolytica* (black bar), *E. invadens* (dark grey bar), and *E. dispar* (white bar) was evaluated with tungstate (T), molybdate (M), and orthovanadate (O), specific PTP inhibitors. All experiments were run with p-NPP as the substrate. Differences between the phosphate activity of the distinct extracts were considered significant at *p* < 0.0001. The data is representative of six independent experiments.

Phosphatase enzymatic activity was analyzed in the total extracts, membrane fractions, and cytosolic fractions of *E. histolytica*, *E. invadens*, and *E. dispar*. A similar level of phosphatase activity was observed in the total extract of all three species. Enzymatic activity detected was in the following hierarchical order: membrane fraction > total extract > cytosolic fraction (Figure 1(a)). To evaluate the percentage of activity by species, phosphatase activity tests were carried out only in the membrane fraction, and we obtained phosphatase activity in *E. histolytica* (100%) compared to the lower relative values for *E. invadens* (88%) and *E. dispar* (56%) (Figure 1(b)).

Subsequently, the corresponding activity in each species was evaluated with a set of inhibitors. The inhibitors that were used were sodium tungstate (T), ammonium molybdate (M), and sodium orthovanadate (O), which specifically inhibit the activity of tyrosine phosphatases.  Figure 1(c) demonstrates that in all three species, similar percentages of inhibition were obtained, ranging from 79% to 94% in all cases.

### 3.2. Detection of PP2C in Trophozoites of *E. histolytica*, *E. invadens*, and *E. dispar*

#### 3.2.1. Western Blot and Immunofluorescence

**Figure 2:**
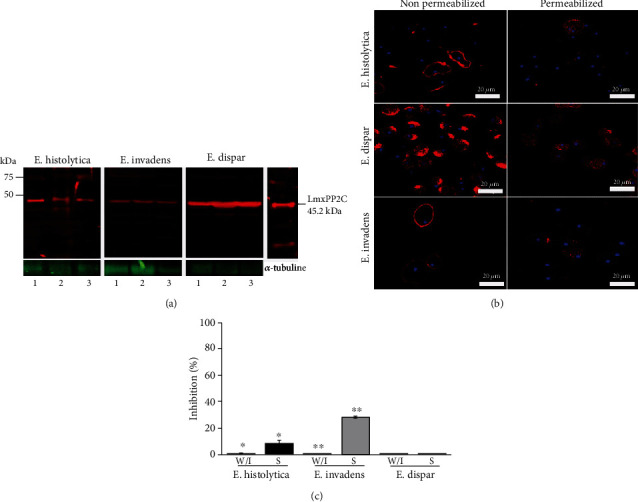
Detection of PP2C in *Entamoeba* spp. (a) Immunodetection of PP2C using the polyclonal antibody against *Lmx*PP2C in the total extract (TE) (lane 1), membrane fraction (MF) (lane 2), and cytosolic fraction (FC) (lane 3). Recombinant protein *Lmx*PP2C was used as a positive control and anti-*α*-tubulin as the loading control. The signal was analyzed on the Odyssey Infrared Imaging System. (b) Immunofluorescence assay using polyclonal antibody against *Lmx*PP2C. Scale bars = 20 *μ*m. (c) Phosphatase activity on the MF of *E. histolytica* (black bar), *E. invadens* (grey bar), and *E. dispar* found by using sanguinarine, a specific inhibitor for PP2C. *p* < 0.05.

An antibody against *L. mexicana* PP2C (anti-*Lmx*PP2C) was utilized to detect PP2C in the total extract, membrane fraction, and cytosolic fraction of *E. histolytica*, *E. invadens*, and *E*. *dispar*. In *E. histolytica*, *E. invadens*, and *E. dispar*, a 45.2 kDa molecule was recognized in all fractions (total extract, lane 1; membrane fraction, lane 2; and cytosolic fraction, lane 3). The 45.2 kDa molecule had a molecular weight like that of the recombinant protein PP2C of *L*. *mexicana* (*Lmx*PP2C, the control). Likewise, the anti-*Lmx*PP2C antibody also recognized a high molecular weight component, which stood out in the case of *E. invadens*. The image is representative of five independent assays. The anti-*α*-tubulin antibody was the load control (Figure 2(a)).

To analyse the subcellular location of PP2C, confocal microscopy assays were performed; in *E. histolytica*, *E. invadens*, and *E. dispar*, localization was evident on the surface of the trophozoites. This localization highlights a differential pattern, since in *E. dispar*, a polarized location and patches were observed on the surface of the nonpermeabilized trophozoites. Under permeabilized conditions, in all cases, it was possible to identify the protein in the cytosol, homogeneously, and in small vesicles. These images were representative of five independent assays (Figure 2(b)).

#### 3.2.2. Inhibition of PP2C Phosphatase Activity in the Membrane Fraction of *E. histolytica*, *E. invadens*, and *E. dispar*

An evaluation was made of the impact of sanguinarine, a specific inhibitor of PP2C protein serine/threonine phosphatase, and we found that sanguinarine exhibited a limited inhibitory effect on phosphatase activity in *E. histolytica* (8%) and *E. invadens* (28%) and none in *E. dispar* (Figure 2(c)). Data represent six independent assays.

These analyses suggest the presence of the protein phosphatase PP2C in the *Entamoeba* species.

### 3.3. Localization of PP2C in *E. invadens* Cysts by Immunofluorescence and Western Blot

**Figure 3:**
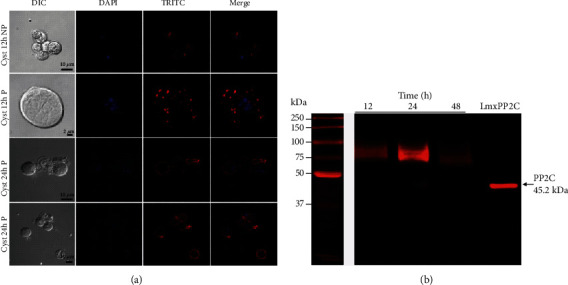
Detection of PP2C in the encysting process of *E. invadens*. (a) Differential Interference Contrast (DIC) column shows the purity of the cysts under permeabilized (P) and nonpermeabilized (NP) conditions at 12 and 24 h after encystment. DAPI was used to show the nucleus; presence of PP2C is observed in red using the anti-*Lmx*PP2C antibody evidenced by secondary antibody coupled to TRITC. (b) Western blot analysis using anti-*Lmx*PP2C antibody on total cyst extracts solubilized with triton. Recombinant protein *Lmx*PP2C was used as a positive control. Scale bars = 10 and 2 *μ*m. The proteins were analyzed on the Odyssey Infrared Imaging System.

The images of *E. invadens* cysts are portrayed in Figure 3(a). At 12 h, the phosphatase protein was observed in the cytosolic fraction of the cyst in a granular pattern (P, in TRITC and MERGE). At 24 h, it was in both the membrane and the cytosol of the cyst (P and NP, TRITC, and merge). To identify PP2C in cysts, immunoblotting assays were performed using the anti-*Lmx*PP2C antibody in membrane and cytosolic fractions obtained from extraction with Triton X-100. A 75 kDa protein was identified mainly in the cytosolic fraction, during the encysting process (at 12, 24, and 48 h). However, in some assays, it was possible to detect the 75 kDa protein in the membranal fraction. We consider that these findings may be the result of processing asynchronous cultures (data not shown). Another possibility is that the 75 kDa protein is very sensitive to proteolytic activity, so it would be necessary to explore with another type of inhibitor's cocktail. In Figure 3(b), a representative image of two tests performed independently shows the results corresponding to the cytosolic fraction. Recombinant *Lmx*PP2C served as the control (Figure 3(b)).

### 3.4. Hypothetical Model of the Presence of PP2C in the Genus *Entamoeba* and Possible Participation of the Enzyme in the Encystment of *E. invadens*

**Figure 4:**
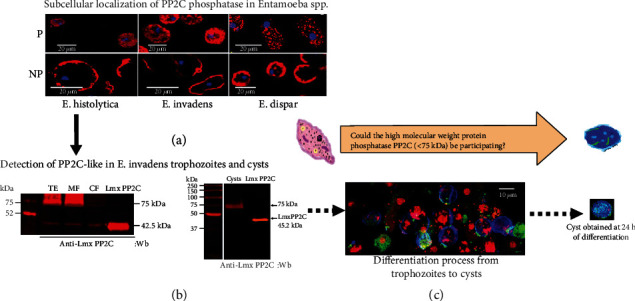
PP2C protein phosphatase expression model in *Entamoeba* spp. (a) Localization of protein phosphatase PP2C in trophozoites. (b) Detection of PP2C in *E. invadens* trophozoites and cysts by Western blot assays. (c) Immunofluorescence of PP2C in *E. invadens* encystation. A specific differential staining was used for cysts, calcofluor (blue), propidium iodide (purple) for the cyst nuclei, and FITC (fluorescein isothiocyanate, green) fluorophore.

The evidence obtained in the present work has shown that the genus *Entamoeba* possesses PP2C with antigenic similarity to the protein PP2C. The anti-*Lmx*PP2C antibody showed subcellular localization both in the membrane and in the small vesicular structures (Figures 4(a) and 4(b)), while in the case of the Western blot assays, the anti-*Lmx*PP2C antibody detected a 45.2 kDa protein and another higher molecular weight protein of approximately 75 kDa, mainly in the encyst (Figure 4(c)). In this context, we propose that this high molecular weight protein possibly participates in the encysting process. The immunofluorescence assays analyzed by confocal microscopy using anti-*Lmx*PP2C corroborate the presence of PP2C in the cystic phase (Figure 4). Given that immunofluorescence tests determined that PP2C participates in the encysting process, in silico analysis was carried out to determine the similarity with the high molecular weight PP2C reported for *E. invadens*. The in silico analysis supported the identity of the high molecular weight component (75 kDa), which could be the PP2C identified in the *E. invadens* genome database.

### 3.5. *In Silico* Analysis

**Figure 5:**
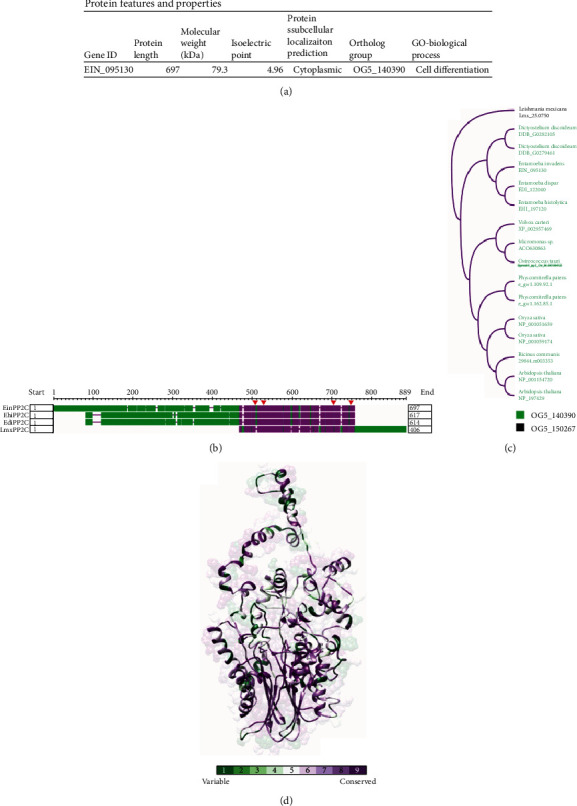
In silico analysis of the PP2C phosphatase protein in *E. invadens* (EinPP2C). (a) Table of properties and characteristics of the *Ein*PP2C sequence (EIN_095130). (b) Graphical summary of the alignment of the sequences of the PP2C proteins of *E. invadens* (*Ein*PP2C, EIN_095130), *E. histolytica* (*Ehi*PP2C, EHI_197120), *E. dispar* (*Edi*PP2C, EDI_122040), and *L. mexicana* (*Lmx*PP2C, Lmx_25.0750), examined on COBALT. The PP2C of *E. invadens* is found at positions D458, D479, D648, and D687 (red arrows). The most conserved region of the PP2C domain is depicted in purple. (c) A cladogram is displayed of the sequences of the proteins in the OG5_150267 orthologues group and the sequence of LmxPP2C (OG5_150267). (d) Analysis of the degree of evolutionary conservation was carried out, comparing the hypothetical 3D structure of EinPP2C with the orthologue sequences of the two other species of *Entamoeba* ssp. and with *L. mexicana Lmx*PP2C, scrutinized on the ConSurf server and visualized on the UCSF Chimera program. The *β*-sandwich domain (in purple) is the most conserved region of the sequences.

An *in silico* search was conducted to identify the 75 kDa PP2C protein encountered in cysts (Figure 3(b)) of *E. invadens*. Considering the characteristics of molecular weight, cell location, and homology with *Lmx*PP2C, the sequence most likely to correspond to the 75 kDa protein is the EIN_095130 gene (in AmoebaDB). The respective protein is characterized by a molecular weight of 79.3 kDa and a cytoplasmic cell location. Additionally, it is part of the OG5_140390 group of orthologues (Figure 5(a)), which at the present time includes fifteen sequences belonging to the protozoan genera *Entamoeba* and *Dictyostelium* and to some plants (e.g., *Arabidopsis thaliana* and *Oryza sativa*).

All these sequences were classified into two main clades according to their phylogenetic group. *L. mexicana* is considered an independent clade because the *Lmx*PP2C protein sequence is encoded by a paralogous gene (Figure 5(c)). A graphical summary is provided (Figure 5(b)) of the multiple sequence alignments between the PP2Cs of *E. invadens* (*Ein*PP2C, EIN_095130), *E*. *histolytica* (*Ehi*PP2C, EHI_197120), *E. dispar* (*Edi*PP2C, EDI_122040), and *L. mexicana* (*Lmx*PP2C, *Lmx*_25.0750). Each protein sequence conserves the four aspartate amino acids implicated in enzymatic catalysis. In the case of *E. invadens*, they were found at positions D458, D479, D648, and D687. The PP2C domain was in the C-terminus of the protein in amoebic species and in the N-terminus of the protein in *L. mexicana*, suggesting distinct functions. An analysis of evolutionary conservation was performed in relation to the structure of the amino acids of the PP2Cs of the amoebic species and the PP2C of *L. mexicana*. At the base of the model (Figure 5(d)), the most conserved region of the PP2C domain is depicted in purple. It should be noted that despite the low amino acid sequence identity between *Entamoeba* and *Leishmania* PP2Cs, it is well known that apparently different antigens within the same protein family can substantially crossreact due to similar 3D structural regions.

## 4. Discussion

The existence of phosphatases in different organisms has been well documented. In 1999, a calcium-dependent phosphatase, calmodulin, was detected in the *Leishmania* parasite by means of crossreaction assays. These experiments were carried out with an antibody designed for a PP2B protein of the human brain that has calcium-dependent phosphatase-calmodulin activity [43]. There are also reports on the phosphatomes of various parasites, such as the trypanosomatids [44], *Plasmodium falciparum* [45], and *E. histolytica* [24]. In the case of the *Leishmania* genus, it has been reported that the PP5 phosphatase participates during metacyclogenesis, maintaining the homeostasis and virulence of HSP83 phosphorylation. [46]. A comparative analysis of the protozoan parasite phosphatome demonstrated that no correlation exists between phosphatase activity and the number of respective proteins in *E*. *histolytica*. Although no information was found in the literature on the phosphatome of *E. invadens*, the genetic families of this species have been described and include phosphatases in 74 uncharacterized genomes [8].

In the present study, the greatest activity of acid phosphatases was exhibited by the membrane fraction (versus the cytosolic fraction or the total extract) of *E. histolytica*, *E. invadens*, and *E. dispar*, even though the highest number of proteins was encountered in the total extract. Of these three species, acid phosphatase activity was greatest in *E. histolytica*. Likewise, it was previously documented that pathogenic *E. histolytica* has a high level of acid phosphatase activity, while nonpathogenic *E. dispar* is unable to generate such activity despite having an abundance of membrane acid phosphatases. Here, insights were provided by separating the distinct types of trophozoite cell extracts (the total extract, membrane fraction, and cytosolic fraction) of *E. histolytica*, *E. invadens*, and *E. dispar* and determining the phosphatase activity in each extract. For example, the level of activity of the acid phosphates did not correlate with their expression level. Additionally, the acid phosphatase activity did not correlate with virulence. Hence, other differences must exist between virulent and avirulent strains that could explain the capacity of the former to trigger disease [47].

In many studies, specific inhibitors of protein phosphatases have been used for the preliminary characterization of those proteins [48–50]. In this study, specific inhibitors of both PTP and the protein serine/threonine phosphatase PP2C were applied to the extracts of the three species of *Entamoeba*. The enzymatic activity in the membrane fraction of the three species evaluated was inhibited by all PTP inhibitors and, to a lesser extent, by sanguinarine (a specific inhibitor of PP2C). This raises many questions about the properties and functions of the PP2C herein identified.

In the present study, a 45.2 kDa molecule was recognized by the anti-*Lmx*PP2C antibody in the total extract, membrane fraction, and cytosolic fraction of *E. histolytica*, *E. invadens*, and *E. dispar*. PP2Cs of the same molecular weight have been identified in other microorganisms [30, 51, 52]. The location of PP2C was confirmed by immunofluorescence, revealing membrane and cytosolic expression in all *Entamoeba* species. The cytosolic and membranal expression of PP2C was similar in *E. invadens* trophozoites, while there was a polarized location in the membranal fraction versus cytosolic expression in *E. dispar* trophozoites.

The activity of acid phosphatases was determined in the trophozoites of *Entamoeba* parasites [53]. That research was conducted prior to the development of cell fractionation and differential centrifugation. The type of phosphatase found in this study was not detected in previous investigations. Indeed, to our knowledge, no previous report exists on PP2C phosphatases in *Entamoeba* species. In the current study, a polyclonal antibody capable of recognizing the PP2C phosphatase localized in the membrane and cytosolic fractions of *E. histolytica*, *E. invadens*, and *E. dispar*.

In *Toxoplasma gondii*, a PP2C regulates the infective process as well as the development of the parasite [54]. In *Leishmania*, metal-dependent phosphatases have been described and are known to be differentially expressed in distinct stages of the parasite. In the case of *Leishmania major*, the flagellar pocket of the parasite contains a PP2C, and recently, a protein serine/threonine phosphatase type 2C was identified and localized in the flagellum and flagellar pocket of promastigotes and amastigotes of *L. mexicana* [30, 52]. In *Plasmodium*, a PP2C regulates the development of the parasite [55]. Recently, identification has been made by immunofluorescence by crossreaction with an antibody of PP2C from *L. major* in *Cryptosporidium parvum* (CpPP2C), observed in the nucleus of oocysts and at the apical end of the sporozoite body [51].

PP2C has been more widely studied in plants, where it is reportedly related to stress. For example, a gene coding for PP2C in *Arabidopsis thaliana* is involved in processes such as germination and osmotic stress, and its expression is induced by abscisic acid [56, 57]. A PP2C phosphatase active was identified and localized in the membrane and cytosolic fractions of trophozoites in *E. histolytica*, *E. dispar*, and *E. invadens* and in cysts of *E. invadens*. Fluorescence showed a granular pattern in the expression of this protein in the trophozoites of *E. dispar* and at 12 h induced cysts of *E. invadens*; this suggests that PP2C phosphatase intervenes in the maturation process of the cyst which is a stress-dependent process. This subcellular location could be involved in the invasive process when pathogenic trophozoites confront target cells and dephosphorylate the receptors and/or other proteins anchored in the membrane of these cells. Due to dephosphorylation, host cells are obliged to reorganize their cytoskeleton, leaving them more vulnerable to being lysed by the parasite. The presence of phosphatases in the cytoplasm of other species may indicate their participation in a translocation process towards the membrane, or in a secretion process. These possibilities, beyond the scope of the current research, should be analyzed in the future. [21].

A comparative in silico analysis of phosphatases has been carried out in a variety of protozoan parasites, showing that serine/threonine phosphatases constitute the highest proportion of all protein phosphatases as well as among those on the plasma membrane [58]. Serine phosphatases are known to take part in L-serine biosynthetic pathways in *E. histolytica* and *E. invadens* [59]. In this study, protein serine/threonine phosphatases were localized on the membrane and in the cytosol of both the trophozoites and immature cysts of *E. invadens* during the process of encystation. Thus, these proteins might be implicated in the transformation of the parasite from one stage to another. Additionally, a 75 kDa cytosolic protein was presently identified in its active form on *E. invadens*, being equally expressed in trophozoites and cysts. Our recent identification and localization of PP2C in different protozoan parasites, such as *Leishmania* and *Cryptosporidium*, open a new perspective on the participation of these enzymes in essential functions of these parasites [30, 51, 52]. The encysting process of *E. invadens* was examined in the current contribution, finding that the PP2C phosphatase is in trophozoites of a molecular size of 45.2 kDa as well as cysts (at different maturation times) of a molecular size of approximately 75 kDa, primarily in the cytosolic fraction. This information suggests a potential role of PP2C in the transformation process of the parasite. The protein phosphatase expression model in *Entamoeba* shows the presence of PP2C.

According to the OrthoMCL database, the PP2C protein of *E. invadens* belongs to the OG5_140390 orthologue group. The orthologous proteins of the most studied member of this group, *Arabidopsis thaliana*, have been characterized as kinase-associated protein phosphatases (KAPPs). One such protein is located on the plasma membrane and in intracellular vesicles that control the internalization of somatic embryogenesis receptor kinase 1 (AtSERK1) [60]. Data found in the *amoebadb* database indicate that EIN_095130 is expressed in immature cysts (before 24 h of induction of the process) [8]. Therefore, PP2C may participate in cell differentiation during the process of encystation of *E*. *invadens*.

One of the principal objectives of research on parasites of medical importance is to find therapeutic targets, these being key molecules in the organisms that participate in the infective process. The protein phosphatase PP2C of *Entamoeba* has been reported to participate in the process of stress in plants and was presently identified in both phases (trophozoites and cysts) of the parasite. The conversion of *Entamoeba* trophozoites to cysts is considered a stress mechanism. The main PP2C expressed is of 75 kDa in cysts, which may be an important target in *Entamoeb*a. Performing tests with sanguinarine, a specific inhibitor of PP2C, will allow for the determination of the participation of the phosphatase during the invasive process of *E. histolytica*. Likewise, the mutation of the PP2C gene that encodes the 75 kDa protein in *E. invadens* and the use of the specific PP2C inhibitor could confirm the participation of this phosphatase during the process of trophozoite encystation.

## 5. Conclusions

The current finding, of PP2C in *E. histolytica*, *E. invadens*, and *E. dispar*, has not been previously reported. The molecule differed in expression, activity, and ultrastructural localization in the two stages (trophozoites and cysts) of the life cycle of *E*. *invadens*. Since phosphorylation and dephosphorylation of the serine/threonine protein control various functions of host-parasite interactions, the *Entamoeba* PP2C identified in this work could be involved in invasion, intracellular replication, and/or the process of encystation of trophozoites, which suggests its potential as a therapeutic target to treat amoebiasis.

## Data Availability

The data used to support the findings of this study are available from the corresponding author upon request.

## References

[1] Ralston K. S., Petri W. A. (2011). Tissue destruction and invasion by _Entamoeba histolytica_. *Trends in Parasitology*.

[2] Marie C., Petri W. A. (2014). Regulation of virulence ofEntamoeba histolytica. *Annual Review of Microbiology*.

[3] Cheepsattayakorn A., Cheepsattayakorn R. (2014). Parasitic pneumonia and lung involvement. *BioMed Research International*.

[4] World Health Organization (1997). Amoebiasis. *Weekly Epidemiological Record*.

[5] Stanley S. L. (2003). Amoebiasis. *Lancet*.

[6] Wu N., Freiman J. S. (2017). Caecal ulceration in an asymptomatic man. *Gut*.

[7] Meerovitch E. (1958). A new host of Entamoeba invadens RODHAIN, 1934. *Canadian Journal of Zoology*.

[8] Ehrenkaufer G. M., Weedall G. D., Williams D. (2013). The genome and transcriptome of the enteric parasite Entamoeba invadens, a model for encystation. *Genome Biology*.

[9] Diamond L. S., Clark C. G. (1993). A redescription of Entamoeba histolytica Schaudinn, 1903 (Emended Walker, 1911) separating it from Entamoeba dispar Brumpt, 1925. *The Journal of Eukaryotic Microbiology*.

[10] Ximénez C., Cerritos R., Rojas L. (2010). Human amebiasis: breaking the paradigm?. *International Journal of Environmental Research and Public Health*.

[11] Talamás-Lara D., Chávez-Munguía B., González-Robles A. (2014). Erythrophagocytosis in Entamoeba histolytica and Entamoeba dispar: a comparative study. *BioMed Research International*.

[12] Jacobs T., Bruchhaus I., Dandekar T., Tannich E., Leippe M. (1998). Isolation and molecular characterization of a surface-bound proteinase of Entamoeba histolytica. *Molecular Microbiology*.

[13] Kato K., Makiuchi T., Cheng X., Tachibana H. (2017). Comparison of hemolytic activity of the intermediate subunit of Entamoeba histolytica and Entamoeba dispar lectins. *PLoS One*.

[14] Wilson I. W., Weedall G. D., Hall N. (2012). Host-parasite interactions in Entamoeba histolytica and Entamoeba dispar: what have we learned from their genomes?. *Parasite Immunology*.

[15] Petri W. A., Haque R., Mann B. J. (2002). The bittersweet interface of parasite and host: lectin-carbohydrate interactions during human invasion by the ParasiteEntamoeba histolytica. *Annual Review of Microbiology*.

[16] Leippe M., Bruhn H., Hecht O., Grötzinger J. (2005). Ancient weapons: the three-dimensional structure of amoebapore A. *Trends in Parasitology*.

[17] Gilchrist C. A., Houpt E., Trapaidze N. (2006). Impact of intestinal colonization and invasion on the _Entamoeba histolytica_ transcriptome. *Molecular and Biochemical Parasitology*.

[18] Barford D. (1996). Molecular mechanisms of theprotein serine/threonine phosphatases. *Trends in Biochemical Sciences*.

[19] Aguirre-Garcia M. M., Rosales-Encina J. L., Talamas-Rohana P. (1997). Secreted Entamoeba histolytica acid phosphatase (SAP). *Archives of Medical Research*.

[20] Anaya-Ruiz M., Pérez-Santos J. L. M., Talamás-Rohana P. (2003). An ecto-protein tyrosine phosphatase of _Entamoeba histolytica_ induces cellular detachment by disruption of actin filaments in HeLa cells. *International Journal for Parasitology*.

[21] Aguirre-García M. M., Anaya-Ruiz M., Talamás-Rohana P. (2003). Membrane-bound acid phosphatase (MAP) fromEntamoeba histolyticahas phosphotyrosine phosphatase activity and disrupts the actin cytoskeleton of host cells. *Parasitology*.

[22] Herrera-Rodríguez S. E., Baylón-Pacheco L., Talamás-Rohana P., Rosales-Encina J. L. (2006). Cloning and partial characterization of _Entamoeba histolytica_ PTPases. *Biochemical and Biophysical Research Communications*.

[23] Ramírez-Tapia A. L., Baylón-Pacheco L., Espíritu-Gordillo P., Rosales-Encina J. L. (2015). Characterization of the protein tyrosine phosphatase PRL from _Entamoeba histolytica_. *Experimental Parasitology*.

[24] Anwar T., Gourinath S. (2013). Analysis of the protein phosphotome of Entamoeba histolytica reveals an intricate phosphorylation network. *PLoS One*.

[25] Diamond L. S., Harlow D. R., Cunnick C. C. (1978). A new medium for the axenic cultivation of Entamoeba histolytica and other entamoeba. *Transactions of the Royal Society of Tropical Medicine and Hygiene*.

[26] Diamond L. S., Clark C. G., Cunnick C. C. (1995). YI-S, a casein free medium for axenic cultivation of Entamoeba histolytica, related Entamoeba, Giardia intestinalis and Trichomonas vaginalis. *The Journal of Eukaryotic Microbiology*.

[27] Sanchez L., Enea V., Eichinger D. (1994). Identification of a developmentally regulated transcript expressed during encystation of _Entamoeba invadens_. *Molecular and Biochemical Parasitology*.

[28] Dissing J., Dahl O., Svensmark O. (1979). Phosphonic and arsonic acids as inhibitors of human red cell acid phosphatase and their use in affinity chromatography. *Biochimica et Biophysica Acta (BBA) - Enzymology*.

[29] Aburai N., Yoshida M., Ohnishi M., Kimura K. (2010). Sanguinarine as a potent and specific inhibitor of protein phosphatase 2Cin Vitroand induces ApoptosisviaPhosphorylation of p38 in HL60 cells. *Bioscience, Biotechnology, and Biochemistry*.

[30] Escalona-Montaño A. R., Zúñiga-Fabián M., Cabrera N. (2021). Protein serine/threonine phosphatase type 2C of Leishmania mexicana. *Frontiers in Cellular and Infection Microbiology*.

[31] Montfort I., Pérez-Tamayo R., Pérez-Montfort R., González Canto A., Olivos A. (1994). Purification and immunologic characterization of a 30-kDa cysteine proteinase of Entamoeba histolytica. *Parasitology Research*.

[32] Martínez-Higuera A., Herrera-Martínez M., Chávez-Munguía B. (2015). _Entamoeba invadens_ : Identification of a SERCA protein and effect of SERCA inhibitors on encystation. *Microbial Pathogenesis*.

[33] López-Contreras L., Hernández-Ramírez V. I., Herrera-Martínez M. (2017). Structural and functional characterization of the divergent Entamoeba Src using Src inhibitor-1. *Parasites & Vectors*.

[34] Herrera-Martínez M., Hernández-Ramírez V. I., Lagunes-Guillén A. E., Chávez-Munguía B., Talamás-Rohana P. (2013). Actin, RhoA, and Rab11 participation during encystment in Entamoeba invadens. *BioMed Research International*.

[35] Aurrecoechea C., Barreto A., Brestelli J. (2011). AmoebaDB and MicrosporidiaDB: functional genomic resources for Amoebozoa and Microsporidia species. *Nucleic Acids Research*.

[36] Horton P., Park K. J., Obayashi T. (2007). WoLF PSORT: protein localization predictor. *Nucleic acids research*.

[37] Papadopoulos J. S., Agarwala R. (2007). COBALT: constraint-based alignment tool for multiple protein sequences. *Bioinformatics. (Oxford, England)*.

[38] Kumar S., Stecher G., Tamura K. (2016). MEGA7: molecular evolutionary genetics analysis version 7.0 for bigger datasets. *Molecular Biology and Evolution*.

[39] Letunic I., Bork P. (2019). Interactive Tree Of Life (iTOL) v4: recent updates and new developments. *Nucleic Acids Research*.

[40] Yang J., Yan R., Roy A., Xu D., Poisson J., Zhang Y. (2015). The I-TASSER suite: protein structure and function prediction. *Nature Methods*.

[41] Pettersen E. F., Goddard T. D., Huang C. C. (2004). UCSF Chimera-a visualization system for exploratory research and analysis. *Journal of Computational Chemistry*.

[42] Ashkenazy H., Abadi S., Martz E. (2016). ConSurf 2016: an improved methodology to estimate and visualize evolutionary conservation in macromolecules. *Nucleic acids research*.

[43] Banerjee C., Sarkar D., Bhaduri A. (1999). Ca2+ and calmodulin-dependent protein phosphatase from Leishmania donovani. *Parasitology*.

[44] Brenchley R., Tariq H., McElhinney H. (2007). The TriTryp phosphatome: analysis of the protein phosphatase catalytic domains. *BMC Genomics*.

[45] Wilkes J. M., Doerig C. (2008). The protein-phosphatome of the human malaria parasite Plasmodium falciparum. *BMC Genomics*.

[46] Norris-Mullins B., Krivda J. S., Smith K. L., Ferrell M. J., Morales M. A. (2018). Leishmania phosphatase PP5 is a regulator of HSP83 phosphorylation and essential for parasite pathogenicity. *Parasitology Research*.

[47] Talamás-Rohana P., Aguirre-García M. M., Anaya-Ruiz M., Rosales-Encina J. L. (1999). _Entamoeba dispar_ Contains but Does Not Secrete Acid Phosphatase as Does _Entamoeba histolytica_. *Experimental Parasitology*.

[48] Zhang Z. Y., Van Etten R. L. (1990). Purification and characterization of a low-molecular-weight acid phosphatase-- A phosphotyrosyl-protein phosphatase from bovine heart. *Archives of Biochemistry and Biophysics*.

[49] Hardie D. G. (1990). Roles of protein kinases and phosphatases in signal transduction. *Symp Soc Exp Biol.*.

[50] Zhang Z. Y., Dixon J. E. (2006). Protein tyrosine phosphatases: mechanism of catalysis and substrate specificity. *Advances in Enzymology - and Related Areas of Molecular Biology*.

[51] Gómez-Sandoval J. N., Okhuysen P., Mondragón-Flores R., Escalona-Montaño A. R., Aguirre-García M. M. (2020). Cellular identification and in silico characterization of protein phosphatase 2C (PP2C) of Cryptosporidium parvum. *Acta Parasitologica*.

[52] Escalona-Montaño A. R., Pérez-Montfort R., Cabrera N. (2017). Protein phosphatase PP2C in the flagellum of Leishmania major: cloning and characterization. *Parasitology Open*.

[53] Aguirre-García M. M., Cerbón J., Talamás-Rohana P. (2000). Purification and properties of an acid phosphatase from _Entamoeba histolytica_ HM-1:IMSS. *International Journal for Parasitology*.

[54] Gilbert L. A., Ravindran S., Turetzky J. M., Boothroyd J. C., Bradley P. J. (2007). Toxoplasma gondii targets a protein phosphatase 2C to the nuclei of infected host cells. *Eukaryotic Cell*.

[55] Zhang M., Mishra S., Sakthivel R., Fontoura B. M., Nussenzweig V. (2016). UIS2: a unique phosphatase required for the development of Plasmodium liver stages. *PLOS Pathogens*.

[56] Schweighofer A., Hirt H., Meskiene I. (2004). Plant PP2C phosphatases: emerging functions in stress signaling. *Trends in Plant Science*.

[57] Zhang J., Li X., He Z. (2013). Molecular character of a phosphatase 2C (PP2C) gene relation to stress tolerance in Arabidopsis thaliana. *Molecular Biology Reports*.

[58] Anwar T., Gourinath S. (2016). Deep insight into the phosphatomes of parasitic protozoa and a web resource ProtozPhosDB. *PLoS One*.

[59] Chiba Y., Makiuchib T., Jeelanib G., Nozakia T. (2016). Heterogeneity of the serine synthetic pathway in *Entamoeba* species. *Molecular & Biochemical Parasitology*.

[60] Shah K., Russinova E., Gadella T. W., Willemse J., De Vries S. C. (2002). The Arabidopsis kinase-associated protein phosphatase controls internalization of the somatic embryogenesis receptor kinase 1. *Genes & development*.

